# Evaluating the Impact of a Simulation Based Training Course in Intellectual Disability Psychiatry and Autism Co-Delivered by Actors With Intellectual Disability

**DOI:** 10.1192/bjo.2022.120

**Published:** 2022-06-20

**Authors:** Sonya Rudra, Rupal Dave, Nicole Eady, James Smith, Eileen McNamara

**Affiliations:** 1 East London NHS Foundation Trust, London, United Kingdom; 2 North East London NHS Foundation Trust, London, United Kingdom; 3 Sussex Partnership NHS Foundation Trust, West Sussex, United Kingdom; 4 Barnet Enfield and Haringey NHS Foundation Trust, London, United Kingdom

## Abstract

**Aims:**

Inequalities in health outcomes, and avoidable deaths, in people with intellectual disability has highlighted the need for improved training and education in Intellectual Disability and Autism. Simulation training facilitated by actors with intellectual disability has been shown to improve connection with people with intellectual disability (Attoe et al 2017). The aim of this project was to develop a simulation-based training course, focused on topics in mental health, intellectual disability and autism, to improve participant confidence in clinical knowledge and skills, as well as support leadership and professionalism training. Here we evaluate the impact of the training on participants’ confidence, and the longer-term effect on attitudes and working practice after attendance.

**Methods:**

A novel simulation-based training course, directed at Specialty Trainees, was developed based on the Specialty Training in Learning Disability curriculum. The course was co-delivered by a person with intellectual disability. Participants who attended the simulation training completed general feedback, pre-course and post-course confidence questionnaires and attended a semi-structured group interview at 2 months. Questionnaire data weres analysed using descriptive and inferential statistics. Group interview data were analysed using open & axial coding, and thematic analysis of content. The project was approved by East London NHS Foundation Trust Governance and Ethics Committee for Studies and Evaluations.

**Results:**

Eight psychiatrists participated in the training and completed the pre-course and post-course questionnaires. Independent t-test found significant increase in confidence for all scores from pre-course (M = 6.54, SE0.24) to post-course (M = 7.81, SE = 0.36), t= –2.93 p = 0.01. This included ratings of confidence in knowledge in areas such as mental health legislation, and improved confidence in skills such as communication with families of people with intellectual disability and difficult conversations with senior supervisors. In follow-up interviews we elucidated themes of the importance of supported, structured training opportunities with people with intellectual disability, and the value of connection with peers and supervisors.

**Conclusion:**

Simulation based training in psychiatry, co-delivered with actors with intellectual disability, was reported to be an engaging and enjoyable form of learning. The evaluation suggests such training is effective in increasing trainee confidence in knowledge and skills at the time of training as well as resulting in a lasting change in attitudes after the training. We recommend such training be further developed and delivered at both postgraduate and undergraduate level.

